# How does better regulation shape EU health policy? A case study of unhealthy advertising regulation

**DOI:** 10.1093/eurpub/ckac131.535

**Published:** 2022-10-25

**Authors:** K Lauber, E Brooks

**Affiliations:** Global Health Policy Unit, University of Edinburgh, Edinburgh, UK

## Abstract

**Introduction:**

Better Regulation is a meta-regulatory tool designed to improve regulatory quality and reduce regulatory burden in the development of EU policy. Despite concerns by civil society that its operation may have a chilling effect on regulatory protections, its impact on health policy has not been researched systematically. Using provisions on high fat, sugar and salt (HFSS) food and alcohol advertising to children within the revision of the audiovisual media services directive (AVMSD) as a case study, we explore how the operation of Better Regulation affects EU health policy processes.

**Methods:**

We employ a qualitative process tracing approach based on policy documents, Freedom of Information requests, media reporting, and expert interview data.

**Results:**

After an evaluation in 2016, the revised AVMSD maintained a reliance on self- and co-regulation of alcohol and HFSS food advertising to children, despite significant evidence supportive of statutory measures and pressure from the public health community to strengthen provisions. This result aligns with calls from commercial actors to retain the status quo. Preliminary results indicate that pathways via which Better Regulation guidelines may have contributed to this outcome include, for instance, the structure and approach used in the impact assessment and the related scrutiny process, the design of the consultation strategy, and the evaluation criteria of the AVMSD proposal.

**Conclusions:**

Considering how policymaking infrastructure - as a key political determinant of health - may shape the processes and dynamics underlying decision-making can support those working towards a policy environment which protects human and environmental health. In examining EU alcohol and HFSS food advertising provisions within the AVMSD revision through a focus on Better Regulation processes, we contribute a novel perspective towards explaining how measures to improve regulatory quality interact with actors’ agency to shape policy outputs.

**Key messages:**

• The Better Regulation agenda should be understood as a key political determinant of health at the EU level.

• Understanding the ways in which governance tools such as Better Regulation can shape health policy is important for those engaged in promoting effective and evidence-informed public health action.

